# Global Estimates on Biological Risks at Work

**DOI:** 10.1016/j.shaw.2023.10.005

**Published:** 2023-10-05

**Authors:** Jukka Takala, Alexis Descatha, A. Oppliger, H. Hamzaoui, Catherine Bråkenhielm, Subas Neupane

**Affiliations:** 1 Unit of Health Sciences, Faculty of Social Sciences, Tampere University, Tampere, Finland; 2 Univ Angers, CHU Angers, Univ Rennes, Inserm, EHESP, Irset (Institut de recherche en santé, environnement et travail), UMR_S 1085, IRSET-ESTER, SFR ICAT, CAPTV CDC, Angers, France; 3 Department of Occupational Medicine, Epidemiology and Prevention, Donald and Barbara Zucker School of Medicine, Hofstra, Northwell, USA; 4 Department of Occupational and Environmental Health, Unisanté, University of Lausanne, Lausanne, Switzerland; 5 Labour Administration, Inspection and Occupational Safety and Health Branch, International Labour Office, ILO, Geneva, Switzerland; 6 Formerly Occupational Safety and Health Coordinator, International Labour Standards Department, International Labour Office, ILO, Geneva, Switzerland

**Keywords:** Biosafety, Exposure, Occupational diseases, Organic chemicals, Work environment

## Abstract

**Introduction:**

Biological risks are a major global problem in the workplace. The recent COVID-19 pandemic has highlighted the need for a more comprehensive understanding of the biological risks at work. This study presents data on both communicable infectious biological agents and noncommunicable factors leading to death and disability for the year 2021.

**Methods:**

We followed the methodology established by the International Labour Organization (ILO) in their past global estimates on occupational accidents and work-related diseases. We used relevant ILO estimates for hazardous substances and related population attributable fractions derived from literature, which were then applied to World Health Organization mortality data. The communicable diseases included in the estimates were tuberculosis, pneumococcal diseases, malaria, diarrheal diseases, other infectious diseases, neglected tropical diseases, influenza associated respiratory diseases and COVID-19. Noncommunicable diseases and injuries considered were Chronic Obstructive Diseases (COPD) due to organic dusts, asthma, allergic reactions and risks related to animal contact. We estimated death attributable to biological risk at work and disability in terms of disability adjusted life years (DALYs).

**Results:**

We estimated that in 2022, 550,819 deaths were caused by biological risk factors, with 476,000 deaths attributed to communicable infectious diseases and 74,000 deaths caused by noncommunicable factors. Among these, there were 223,650 deaths attributed to COVID-19 at work. We calculated the rate of 584 DALYs per 100,000 workers, representing an 11% increase from the previous estimate of the global burden of work-related disabilities measured by DALYs.

**Conclusion:**

This is a first update since previous 2007 ILO estimates, which has now increased by 74% and covers most biological risks factors. However, it is important to note that there may be other diseases and deaths are missing from the data, which need to be included when new information becomes available. It is also worth mentioning that while deaths caused by major communicable diseases including COVID-19 are relatively rare within the working population, absences from work due to these diseases are likely to be very common within the active workforce.

## 1 Introduction

Biological hazards are a more significant problem than previously considered. Hazard identification includes an extensive list of biological agents, substances pathogens and processes that could be present in workplaces [1]. Workplace biological hazards including infectious agents negatively affect workers' health, either directly through infection or indirectly by damaging the working environment. Occupations that involve interaction with specific groups, especially infected individuals, carry a higher risk of infection. Those who work with animals are also at risk of contracting zoonotic infections. Biological hazards can include medical waste or samples of microorganisms, viruses, or toxins derived from biological sources. Quantifying the level of various biological risks is crucial to prevent work-related deaths and disabilities. The International Labor Organization (ILO) previously developed a methodology to quantify the level of various risks of hazardous substances and chemicals at work [2,3]. While identifying the magnitude of all possible risks may not be feasible, understanding the severity and frequency of risks caused by biological agents is important to comprehend the burden. Currently, only limited data exist on the level of various biological risks in workplaces, and the prevalence of biological risk is higher in low-income countries, yet it is widely ignored.

Some occupations such as gardeners, waste disposal workers and wood processing workers, are exposed to biological risks causing cancer, such as hepatoma caused by hepatitis virus and stomach cancer in farming. Carcinogenic aflatoxins produced by certain molds can be present in many work environments, including crop and vegetable cultivation, animal farming, waste sectors, wood sectors, and food processing plants. However, cancer diseases caused by biological factors are not fully covered in these estimates; some of them are covered by past and new comparative global estimates [2,3] on all work-related factors. In previous global estimates, selected biological carcinogens, such as those carcinogens related to farming, grain dust, animal contact, aflatoxins, gardeners, hardwood and leather dust, and combined factors, were presented, but attributable fractions are not separately identifiable for them.

Noncommunicable diseases including chronic respiratory conditions such as COPD and asthma can result from occupational exposure to organic dust, including flour, cotton, and animal originated materials. These diseases can arise from various biological sources, such as animal dusts, molds, yeasts, and organic waste. Occupations like farming and baking pose a significant risk due to organic dust exposure, leading to chronic respiratory problems. Furthermore, organic dusts, smoke, bioaerosols, and vapors can cause various other diseases, including wood dust-related ailments and fungal allergies.

Other acute or chronic diseases caused by organic dusts, vapors, such as wood dusts (including sensitization), skin reactions and allergies to organic materials such as fungi can increase risks when combined. Some outcomes of biological risks are classified as accidents in several countries when immediate consequences are present, such as snake/insect bites/needlestick injuries, animal attack/behaviors, etc. Moreover, exposures at work, and all working situations where workers can be exposed to biological hazards, need to be identified, which has not been systematically done until today.

When estimating the exposures, the following areas and jobs should be considered: animal-related and insect-related occupations, such as those in agricultural and animal farming work; abattoir and slaughterhouse workers; food processing work; as well as handling and distribution work. Health care, laboratory and veterinarian work, as along with occupations at risk for needle-stick injuries are waste and wastewater workers, cleaners, workers maintaining air-conditioning systems, and rescue workers. Sex workers, occupations that involve traveling or contact with travelers, as well as service sector occupations, occupations involving exposure to biological agents in other economic sectors, such as construction, child and elderly care, social service work, work in educational institutions, as well as other work involving close proximity with other people [4].

The agents, substances and circumstances related to biological risks are essential to be considered. There are two main groups of biological agents regarded as occupational hazards: allergenic and/or toxic agents forming bioaerosols, and agents causing zoonoses and other infectious diseases. These agents can cause of allergic and/or occupational immune-toxic diseases of the respiratory organs (airways inflammation, rhinitis, toxic pneumonitis, hypersensitivity pneumonitis, and asthma), conjunctivitis, and dermatitis in exposed workers [5]. A recent systematic review provides an updated overview of the evidence on the exposure to pathogens among non-healthcare workers. These pathogens can be easily transmitted in workplaces where employees gather, emphasizing the significance of workplace risk factors. The authors have established a comprehensive table on exposed occupational groups, pathogens and significant risk factors [6]. The Health and Safety Executive (HSE/UK) has also established a full list of approved dangerous pathogens [7], and the European Union has issued an EU Directive 2000/54/EC – biological agents at work (last modified 04/04/2022) [8]. The International Labour Organisation, ILO refers to biological agents and infectious diseases in the ILO List of Occupational Diseases [9] and the International Agency for Research on Cancer, IARC of WHO has listed carcinogens linked to infectious conditions [10]. Furthermore Cook and Farrant refer to biological agents causing various types of Zoonosis [11]. Table 1 shows comprehensive list of the biological agents from global perspectives based on a systematic review [6].

**Table 1:** A list of typical occupation and related biological agents based on Acke et al. [6]. The list of EU Directive, see [8].

Occupation (ISCO code)	Biological agent
Airline personnel (5111)	Hepatitis E virus, Measles morbillivirus, coronavirus
Animal carers (5164)	*Bartonella henselae, Borrelia burgdorferi, Capillaria hepatica, Campylobacter* spp*, Chlamydia psittaci, Coxiella burnetii,* canine H3N8 influenza virus, *Leptospira* spp, Lymphocytic choriomeningitis virus, mouse retroviruses (XMrV/MLV), Simian foamy virus, simian parvovirus, simian type D retrovirus, Toxocara canis, *Toxoplasma gondii*
Archaeologists (211)	*Coccidioides immitis*
Armed forces (0000)	Methicillin-susceptible *S. aureus*, Adenovirus (7/11A/b) Astrovirus, chikungunya virus, *C. pneumoniae*, Coxsackie virus (A6), *C. burnetii*, dengue virus, ECHO virus, hepatitis A/B/C/e virus, Influenza, A(H1N1/H3N2/H1N1pdm09)/B virus, *Legionella* spp, *Leishmania* spp, Measles morbillivirus, *Microsporum canis, Mycobacterium tuberculosis*, Mumps rubulavirus Norovirus, *Orientia tsutsugamushi*, *Plasmodium falciparum/ovale/vivax*, Respiratory syncytial virus Ross river virus, non-typhoidal *Salmonella enteretica* Sapovirus, *Sarcoptes scabiei*, SARS-CoV-2 virus, *Streptococcus pneumoniae, Streptococcus pyogenes, Trypanosoma cruzii, Yersinia enterocolica*, coronavirus
Bar workers (513)	HIV
Barbers (5141)	Hepatitis B virus
Building workers (711)	*Coccidioides immitis* *Histoplasma capsulatum*
Cash collectors (523)	*Mycobacterium tuberculosis*
Civil engineering labourers (9312)	*Legionella pneumophila*
Cleaners (515)	Hepatitis A virus, Hepatitis B virus, *Mycobacterium tuberculosis*
Divers (7541)	*Campylobacter jejuni,* Enteroviruses, *Pseudomonas aeruginosa*
Farm workers, crops (6111)	*Borrelia burgdorferi, Clostridium tetani, Coccidioides immitis, Coxiella burnetii, Escherichia coli, Francisella tularensis, Leishmania spp, Leptospira borgpetersenii*/spp *Strongyloides stercoralis*, Tick-borne encephalitis virus Toscana virus, *Toxocara canis,* Usutu virus, West Nile virus
Firefighters (5411)	*Cryptosporidium parvum*
Fishmongers (7511)	Anasakis simplex, Hepatitis E virus
Forestry workers (6210)	*Anaplasma phagocytophilum, Bartonella henselae* *Borrelia burgdorferi/miyamotoi, Coxiella burnetii, Francisella tularensis,* Hantavirus, Hepatitis E virus *Leptospira* spp, *Rickettsia conorii, Rickettsia helvetica* Tick-borne encephalitis virus, Toscana virus, *Toxoplasma gondii*, Usutu virus, West Nile virus
Gardeners (6113)	*Francisella tularensis*
Hotel workers (9112)	*Legionella pneumophila*
Livestock and dairy producers (6121)	Methicillin-resistant *S. aureus*, Extended spectrum β-lactamase (≈/AmpC-producing E. coli), Equine/swine/avian influenza virus, *B. anthracis* *Brucella* spp, *Campylobacter* spp, *Chlamydia psittaci* *Clostridium tetani*, *Coxiella burnetii*, Crimean-Congo haemorrhagic fever virus, *Helicobacter pylori* Hepatitis E virus, *Leishmania* spp, L., *Icterohaemorrhagiae*/spp, *Mycobacterium bovis* Rift Valley fever virus, *Salmonella* spp, severe fever with thrombocytopenia syndrome virus, *Streptococcus suis* *Strongyloides stercoralis, Toxocara canis, Toxoplasma gondii,* West Nile virus
Livestock farm labourers (9212)	Methicillin-resistant *S. aureus,* multidrug-resistant *S. aureus,* methicillin-resistant coagulase-negative staphylococci, Extended spectrum β-lactamase/AmpC-producing E. coli, STEC O157/non-STEC O157 Avian/swine influenza virus, *Aspergillus flavus* *Aspergillus fumigatus, Borrelia burgdorferi* *Brucella* spp, *Campylobacter* spp, *Candida albicans* *Chlamydia psittaci, Clostridium* spp, *Clostridium tetani* *Coxiella burnetii, Cryptosporidium parvum* *Helicobacter pylori*, Hepatitis E virus, *Leishmania icterohaemorrhagiae, Moraxella* spp, *Mycobacterium bovis* *Prevotella* spp, *Rickettsia conorii, Rickettsia feliz*, Rift Valley fever virus, Non-typhoidal *Salmonella enteretica* *Strongyloides stercoralis, Toxocara canis, Toxoplasma gondii*, West Nile virus
Manicurists (5142)	Hepatitis B virus, Hepatitis C virus, HIV
Mining and mineral processing plant operators (811)	Panton-Valentine leucocidin-producing methicillin, susceptible *S. aureus, Leptospira* spp, Marburg virus Measles morbillivirus, *Mycobacterium tuberculosis, Sporothrix schenckii*
Office clerks (4110)	Mumps rubulavirus
Plant and machine operators and assemblers (metal and textile/leather) (812, 815)	*Bacillus anthracis, Coxiella burnet*

ISCO, International Standard Classification of Occupation.

Furthermore, specific work and circumstances need to be considered, especially in small and medium-sized enterprises, and among vulnerable groups, such as young workers, elderly workers, pregnant workers, immunosuppressed workers, cleaners, and maintenance workers, as well as migrant and temporary workers, platform workers (including self-employed and contract workers), and those in low socio-economic areas facing circumstances of poverty and informal sector. All work environments lacking clean water, sanitation, sewage systems, and hand-washing facilities, including workers in a total population of roughly 2 billion people living in water-stressed countries [12], should be taken into account. It is essential to address emerging risks and their causative factors, and be prepared for, anticipate, and identify new and/or gradually growing and emerging risks, potential pandemics, and risks in sectors of concern, while implementing global warning systems. This means that both communicable and infectious agents and noncommunicable factors leading to death, disability, and disease need to be covered.

An earlier estimate for fatal work-related communicable diseases, done in 2007, was 320,000 deaths [13]. The aim of the study is to update this 2007 estimation and later estimates [2,3] in light of the recent COVID-19 pandemic.

## 2 Methods

We followed the key steps of the methodology used in the past ILO Global Estimates, such as the report released by ILO, International Commission on Occupational Health (ICOH), and the Government of Singapore in 2017 [2], using data from 2014 [2,3], and the latest estimate from the ICOH 2022 Congress [14].

In summary, we collected data on diseases, deaths, and disabilities from various sources, including the World Health Organization (WHO), ILO, and global burden of diseases (GBD) studies, and scientific literature. To estimate the work-relatedness of these data, we used population attributable fractions (PAF), following the practices of past estimates by ILO, WHO and GBD [2]. Existing estimates of PAF were extended to previously non-covered factors. In cases where PAF was not readily available, we sought expert opinions from research institutions and professional bodies, such as ICOH. If no information on PAF was identified, we used proxy values based on likely similar exposure patterns. For example, for virus infections like A- and B-influenza viruses at work, we considered relatively close droplets and aerosol dissemination patterns. However, it is essential to note that the potency of causing a serious disease or epidemics may differ, even if exposure patterns are comparable.

Our analysis determined the total number of deaths resulting from work injuries, including fatal injuries reported to the ILO by member States [3]. Additionally, it examined the total number of deaths caused by work-related diseases. To achieve this, we utilized all-cause mortality data from the WHO, which was further broken down by diseases, groups of diseases, gender, and age groups.

For the computation of disease-specific population attributable fractions (PAFs), we used information on the number of exposed workers and risk ratios (RR). These work-related PAFs were then applied to the all-cause mortality data from 2019. The PAFs mainly draw references from past ILO Global Estimates and research conducted by Hämäläinen et al. [2] and Takala et al. [3]. In some cases, PAFs were updated with more recent data.

To ensure the accuracy and relevance of our analysis, we reviewed the epidemiological studies from which the PAFs were derived, along with the source industries and occupations to which these PAFs were associated. To exclude non-work-related deaths, certain conditions were applied. For example, deaths in children were excluded by considering only deaths within specific age groups. Additionally, ILO sources of employment data were utilized to eliminate the occupationally non-active population from our analysis.

The seriousness of the risks considered the number of mitigating factors and the vulnerability of specific populations to various exposures. For instance, common diarrhea maybe a minor issue in industrialized countries, but it can be a matter of life and death for poor and exposed populations. Moreover, the availability of prevention methods and treatment options varies widely across different parts of the world. We followed models similar to those used for estimating deaths attributed to hazardous substances [2,3].

We presented the number of diseases, disorders, injuries and deaths, as well as economic cost estimates by the most important disease group. While fatalities due to materialized risks are a limited indicator, disabilities in terms of Disability Adjusted Life Years (DALYs) may indicate a more significant problem. Disabilities at work cover not only Years of Life Lost (YLL) but also Years lived with Disability (YLD). We estimated the deaths, disabilities and injuries resulting from communicable diseases at work, including tuberculosis, pneumococcal diseases, malaria, and selected tropical diseases, such as dengue fever and Ebola infections. These diseases are caused by various factors and agents, including bacteria, viruses, COVID-19 and related viruses, SARS, influenza, as well as agents related to hepatitis and other pathogens, living organisms, fungi, animals, and farming. Snakes, insects, needlestick injuries may cause both long-term and immediate outcomes. The estimated magnitude of risks should encompass all possible routes of exposure, such as ingestion (including poor water quality, sanitation and sewage systems), inhalation, and contact with the skin or mucous membranes, etc.

### 2.1 Statistical analysis

We used biological risk factors from the ILO database, and related population attributable fractions derived from literature were applied to WHO mortality data. Mortality data were obtained from the ILO Global Estimates, while YLL were obtained from the ILO death estimates multiplied by the average years of lives lost. We presented the results separately by major communicable, noncommunicable diseases, and injuries globally, covering people at work.

The economic consequences due to occupational exposure to biological risks were calculated through the number of Disability-Adjusted Life Years (DALYs) as a percentage of the total number of work years that could have been produced if no one had been suffering from any diseases, injuries, and deaths caused by these biological risks.

## 3 Results

### 3.1 Deaths

The estimated total number of deaths due to biological risk factors in 2021 is 550,000. However, it's important to note that due to insufficient data for a fair number of diseases, disorders, and injuries, the total number of deaths is likely to be underestimated.

Eliminating those to avoid double counting, there are 313,521 deaths not covered by existing earlier estimates, which include all work-related diseases and animal-related injuries. As a result, biological risk factors cause 9.8% of all the previously estimated work-related deaths, representing an increase of 10.8% from the earlier figure of 2.9 million work-related deaths.

Table 2 provides the details of the estimated deaths caused by diseases, disorders, and injuries. The table classifies the deaths into two main categories: communicable diseases and noncommu-nicable diseases and injuries, represented by dark green rows.

**Table 2:** Estimated deaths attributed to biological hazards at work.

**Diseases and injuries**	**No. of deaths, working age 20–60 years**		**Estimated % attributed to biological hazards**		**No. of deaths attributed to biological hazards**		
	**Men**	**Women**	**Men**	**Women**	**Men**	**Women**	**Total**
***Infectious diseases without COVID-19, and influenza***	***798,062***	***537,504***			***64,424***	***179,519***	***243,943***
Tuberculosis and pneumococcal diseases	499,852	292,749	3.05	20.7	33,500	154,139	187,639
Malaria	80,377	60,186	10.37	10.37	8,335	6,241	14,576
Diarrheal disease	116,048	117,131	10.37	10.37	12,034	12,146	24,180
Other infectious diseases	90,287	53,393	10.37	10.37	9,363	5,537	14,900
Neglected tropical diseases†	11,498	14,045	10.37	10.37	1,192	1,456	2,648
Influenza associated respiratory deaths, m/f	294,000		3.0	3.0	n/a	n/a	8,820
***COVID-19 annual average 2020-21, excess morbidity WHO, m/f combined***	***7,455,000***		***3.0***	***3.0***	***n/a***	***n/a***	***223,650***
**Communicable diseases. Total**		9,084,586		5,24%			**476,413**
***Respiratory diseases***	***2,060,322***	***1,597,439***	***1.83***	***0.7***	***37,700***	***11,200***	***48,900***
Chronic obstructive pulmonary disease by organic dusts, fumes, aerosols	1,855.560	1,366,670	1.8	0.6	33,400	8,200	41,600
Asthma	204,762	230,769	2.1	1.3	4,300	3,000	7,300
***Animal contact venom./non-venom.***	***22,944***	***17,352***	***10.0***	***10.0***	***2,290***	***1,740***	***4,030***
***Animal injuries, extrapolated***‡	***201,272***	***110,778***	***6.9***	***6.9***	***13,852***	***7,624***	***21,477***
**Non-communicable diseases and injuries, total**§	**2,284,538**	**1,725,569**	**2.4**	**1.2**	**53,842**	**20,564**	**74,406**
**Total**			n/a			n/a	**550,819**

† Including Chagas disease, Leishmaniasis, Schistosomiasis, Dengue fever, Yellow fever, Ebola and other NDT's.

‡ Animal-related injuries were based on United States statistics and extrapolated from there.

§ Cancer deaths caused by biological risk factors are not specifically estimated included/available. Selected cancer deaths caused by biological risks are already covered by ***all*** occupational cancer estimates of 2021 rising to 842,800 deaths.

Due to non-availability of work-relatedness fractions of several diseases and injuries caused by biological hazards, specific estimations for deaths related to all these diseases could not be provided. Some of them are partially covered by the overall occupational deaths considered in the Global Estimates [14] produced by ICOH in collaboration with Tampere University and the Ministry of Social Affairs and Health of Finland and reported in Comparative Global Estimates.

Occupational cancer cases may also include diseases caused by biological hazards, but the exact share of work-related cancer attributable to biological hazards is not easily quantifiable due to a lack of specific data. This includes several cancer types (Box 1).

Box 1 **Occupational cancers caused by biological hazards at workplace.**

• Stomach cancer related to farming and rearing of livestock (due to exposure to grain dust and animal contact).
• Liver and intrahepatic bile ducts cancer by aflatoxins found in crops used by the livestock feed-processing industry.
• Pancreas cancer linked to exposures as gardeners (specific agents not detailed).
• Nose and nasal cancer associated with exposure to hardwood dust, softwood dust, or both, as well as leather dust (common in shoe and boot manufacturing).
• Bronchus and lung cancer linked to specific industries or jobs such as wood processing, printing, cleaning services, hairdressing (due to hair dyes and colorants), housekeeping, and waste disposal.
• Female breast cancer associated with working in hairdressing (exposure to hair dyes).
• Ovary cancer linked to exposure to leather dust, and also hairdressing (exposure to hair dyes and colorants).
• Urinary bladder cancer caused by exposure to leather and rubber in the workplace.

Alt-text: Box 1

**Fig. 1:**
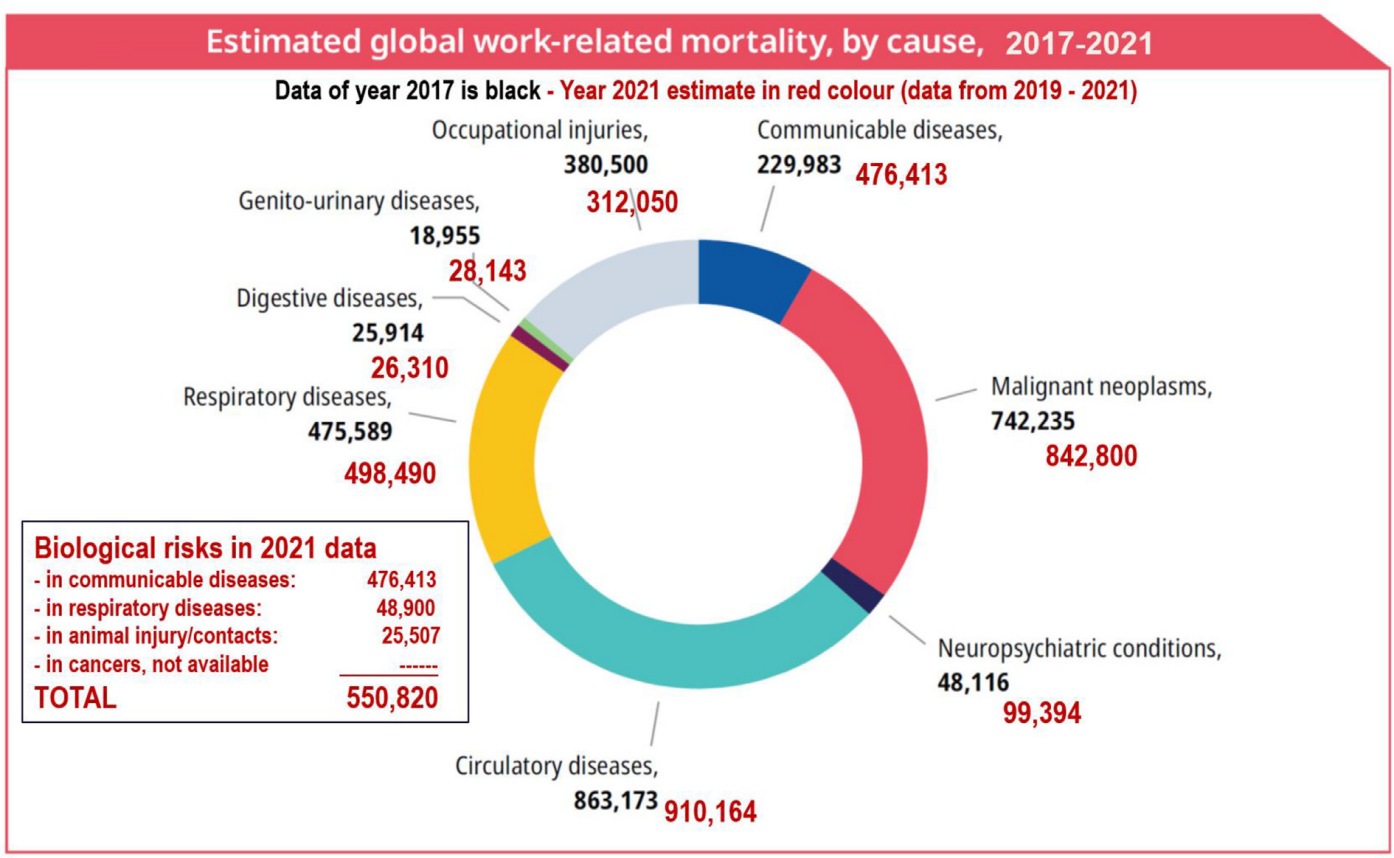
Estimated global work-related morality, by cause, 2017-2021.

Fig. 1 shows the magnitude of the consequences of biological risks in comparison to other work-related risks reported elsewhere [14,15]. As data related to biological hazards was not available when estimating all work-related deaths based on the year 2019, the global total numbers (then 2.9 million deaths) are no longer fully accurate, but they provide an approximate share of the biological risk factors. Work-related communicable disease deaths are included in the injury and respiratory disease sectors of the pie chart, while they are correctly displayed separately in the box dedicated to biological risks.

Other factors and exposures that are poorly identified as work-related and not covered in our estimate include further work-related components [16], such as:

• Hodgkins disease in farmers,
• Cryptogenic fibrosing alveolitis caused by wood dusts,
• Hepatitis A, B, C, D, E among forestry, sewer, pathologists, laboratory staff, healthcare staff, veterinary surgeons,
• Melioidosis in farmers,
• Anthrax from exposures to wool and hides,
• Brucellosis from exposures to livestock,
• Nipah virus in pig farmers, and abattoir workers,
• Livestock-associated MRSA,
• Lyme disease in outdoor workers like forestry workers, hunters, foragers, and others exposed to tick bites [17],
• COPD and asthma related to exposure to organic molds and dusts.

Furthermore, only a limited number of studies exist on the combined effects of several work-related modifiable factors, which are not considered in the table above. It is difficult to estimate the total magnitude of these missing items; however, together they likely form a sizeable component.

#### 3.1.1 Disability and economic costs

Detailed calculations of the DALYs related to exposures to biological hazards at work have not been calculated by disease groups, but an overall estimate indicates that the DALY number increase by approximately the same percentage as death numbers, around 10.8%. Consequently, the global DALY estimate was 5,390 DALYs/100,000 workers without additional biological risk consequences. The additional DALYs caused by biological hazards in this study amount to 584/100,000 workers, which is an additional 0.58 percentage points to be added to the total of 5.8% annual work-related economic loss of the Global Gross Domestic Product (GDP) [14].

The global annual economic cost globally caused by poor prevention of biological diseases and injuries is estimated at 0.58% of the global GDP, which amount to 548 billion USD, or 548 × 10^9^ USD based on the IMF GDP data.

## 4 Discussion

### 4.1 Disease calculations

We estimated biological risks where the information was accessible, and further details can be re-calculated in future work of global estimates on all work-related risk factors. For the year 2021, the largest component was attributed to COVID-19, which significantly increased work-related deaths to 223,000 fatal cases. However, the population attributable fraction for fatal COVID-19 cases was relatively low at 3%, while the fraction of working-age population infected was much higher at 30%. This indicates a significant impact on temporary and long-term disability due to COVID-19, affecting around 19.4% of work-related cases [18].

In 2007, a past estimate recorded 320,000 deaths from communicable diseases [13]. Since then, rapidly developing Asian countries have shown a favorable trend in reducing mortality and morbidity caused by such diseases. However, the rise of occupational hazards and risks from biological factors, especially in low-income countries, has not been adequately addressed from a preventive perspective. In many of these countries, Workers' Compensation Systems coverage remains insufficient or non-existent.

Recognizing the importance of biological hazards at work, the ILO is eager to reassess the negative outcomes, particularly in light of the global impact of the COVID-19 pandemic on workers. The study highlights pandemics as the most significant factor contributing to higher negative outcomes, especially in less developed countries. COVID-19 alone has doubled the negative outcomes compared to past communicable disease estimates. However, so far, there is no clear evidence of the actual impact of occupational exposure on COVID-19 related mortality and morbidity. Previous study from England showed 20%–30% contribution of work exposure on COVID-19 mortality [19], while another Danish study showed increased risk for morbidity in specific occupational groups [20]. Additionally, biological hazards have been classified under other disease groups, such as work-related chronic respiratory diseases and occupational cancer, further contributing to increased negative outcomes. Unfortunately, global compensation systems inadequately recognize occupational exposures as a preventable cause of this burden. The majority of deaths occurs and continues to occur in less developed countries, whereas high-income countries demonstrate a stronger capacity for prevention and successful treatment.

Definitions of varied data sources may cause some relatively small factual error. For example, data on fatalities in Table 1 has been obtained from WHO mortality and GBD studies [21]. Furthermore, the work-related PAFs are from several scientific articles and past estimates [22]. Work-related COVID-19 component in relation to other occupational diseases has been further discussed elsewhere [15].

The Group PAFs for biological risks have been retrospectively calculated from the sum of itemized estimates. However, the magnitude and reporting level related to the number of deaths in original sources, usually government reports, are likely to be higher, causing inaccuracies. In many low-income countries, data does not exist, and in such cases, proxy country sources have been used [23].

The Table 1 also includes some communicable diseases that have been covered in earlier estimates. Thus, one cannot simply add these to the global estimates of 2.9 million work-related deaths based on the source data (year 2019) and released in 2022 [14]. However, two thirds or approximately 313,000 of the total 550,000 deaths have not been estimated with this method so far. Annual variation may be higher for communicable diseases than noncommunicable diseases. The non-identified data in the current estimates means that the total of 550,000 fatal cases is unlikely to be an overestimate.

Based on our calculations, after excluding previously accounted for limited biological factors and related deaths, biological risk factors would contribute to 9.8% of the new total of 3.2 million (2.9 million plus 0.314 million) work-related deaths. This represents a 10.8% increase from the earlier estimate of 2.9 million work-related deaths.

#### 4.1.1 Occupational animal injury

United States statistics provided estimates for occupational animal injuries with an annual number of 375 deaths in the US workforce [24]. Although this data is from 20 years ago, it is likely that the current global death rates are not lower. The animal husbandry sector's significance in the global economy suggests that the global burden of animal injuries is considerably higher.

Data from Bangladesh, covering a population of 1.17 million, reported 1635 animal injuries per100,000, with a rate of 0.7 *fatal* animal injuries per 100,000 [25]. WHO estimated 55,000 fatalities worldwide related to animal injuries at work and outside work.

A rough estimate based on Bangladesh and WHO data [26] suggests global deaths ranging from 14,025 to 28,930. However, the closest estimate is likely around the mid-point of 21,478, after eliminating the non-work-related deaths and considering that the majority of animal handling is done by workers in a low socio-demographic workforce, mainly in Asia and Africa. Moreover, a large number of occupational traffic-related animal injuries should be kept in mind as the same traffic routes are used both for animals and humans—and often mixed in low-income areas.

#### 4.1.2 Limitations

The current estimates have limitations, as some biological risk factors have not been fully estimated due to lack of data sources. These selectively covered problems include tuberculosis, pneumococcal diseases, allergenic/toxic/infectious agents forming bioaerosols, vector-borne zoonoses, other zoonotic agents, tick-borne diseases, mosquito-borne diseases, other infectious non-zoonotic agents, and cancers caused by occupational biological hazards [3]:

• Tuberculosis and Pneumococcal diseases.
• Allergenic, toxic, and infectious agents forming bioaerosols.
• Agents causing vector-borne zoonoses, including emerging or re-emerging diseases of global concern, such as hantaviral diseases, avian and swine influenza, Q fever, leptospirosis, staphylococcal diseases caused by methicillin-resistant *Staphylococcus aureus* (MRSA) strains, and diseases caused by parasitic protozoa.
• Other zoonotic agents, including agents causing allergic and/or immunotoxic occupational diseases of respiratory organ (such as airways inflammation, rhinitis, toxic pneumonitis, hypersensitivity pneumonitis and asthma), conjunctivitis and dermatitis in exposed workers.
• Agents causing tick-borne diseases, such as Lyme borreliosis, anaplasmosis, babesiosis and bartonellosis.
• Exposures to mosquito bites causing malaria, the most prevalent vector-borne disease in the world. However, it is not easy to make a distinction between occupational versus non-occupational exposures, for example, occurring at a wet rice cultivating field, or during free-time or sleep in nearby areas. The selected population attributable fraction, chosen as 10% could also be 80% depending on the selection criteria.
• Other infectious, non-zoonotic agents, where the greatest hazard for health care workers is posed by blood-borne human hepatitis and immunodeficiency viruses (HBV, HCV, HIV, SARS, COVID-19).
• Bacteria causing legionellosis in people occupationally exposed to droplet aerosols, mainly from warm water.
• Cancers caused by occupational biological hazard factors.

## 5 Conclusion

Biological hazards significantly contribute to work-related deaths, disability, and economic losses worldwide. The authors support discussing the need for regulatory and guidance action on “biological hazards in the working environment” in the 112nd Session, 2024 of the International Labour Conference, which is the highest decision-making body of ILO [27]. Measures such as regulatory and control measures, increasing coverage of occupational risks in compensation systems, and strengthening knowledge of prevention measures are vital to enhance prevention, preparedness, and resilience against biological risk factors.

In conclusion, further action is necessary to address biological risk factors effectively, both ethically and economically. The establishment of ““Universal Occupational Health Services” with a strong focus on prevention is essential in this regard.

## Conflicts of interest

The authors declare no conflict of interest.
